# Deep Tendon Reflex: The Tools and Techniques. What Surgical Neurology Residents Should Know

**DOI:** 10.21315/mjms2021.28.2.5

**Published:** 2021-04-21

**Authors:** Ooi Lin-Wei, Leonard Leong Sang Xian, Vincent Tee Wei Shen, Chee Yong Chuan, Sanihah Abdul Halim, Abdul Rahman Izani Ghani, Zamzuri Idris, Jafri Malin Abdullah

**Affiliations:** 1 Department of Neurosciences, School of Medical Sciences, Universiti Sains Malaysia, Kubang Kerian, Kelantan, Malaysia; 2 Neurology Unit, Department of Internal Medicine, School of Medical Sciences, Universiti Sains Malaysia, Kubang Kerian, Kelantan, Malaysia; 3 Hospital Universiti Sains Malaysia, Universiti Sains Malaysia, Kubang Kerian, Kelantan, Malaysia; 4 Department of Neurosurgery, Hospital Umum Sarawak, Kuching, Sarawak, Malaysia; 5 Department of Neurosurgery, Hospital Sungai Buloh, Sungai Buloh, Selangor, Malaysia; 6 Brain Behaviour Cluster, School of Medical Sciences, Universiti Sains Malaysia, Kubang Kerian, Kelantan, Malaysia

**Keywords:** deep tendon reflex, myotatic reflex, muscle spindle, afferent, spinal cord, nervous system, reflex hammer, tapping force, reflex torque, contraction time, relaxation time, striking point, peripheral nerve, effector, technique, biceps reflex, brachioradialis reflex, knee jerk, Achilles reflex

## Abstract

The deep tendon reflex (DTR) is a key component of the neurological examination. However, interpretation of the results is a challenge since there is a lack of knowledge on the important features of reflex responses such as the amount of hammer force, the strength of contraction, duration of the contraction and relaxation. The tools used to elicit the reflexes also play a role in the quality of the reflex contraction. Furthermore, improper execution techniques during the DTR assessment may alter the findings and cloud the true assessment of the nervous system. Therefore, understanding the basic principles and the key features of DTR allows for better interpretation of the reflex responses. This paper discusses the brief history of reflexes, the development of the reflex hammer, and also the key features of a reflex response encompassing the amplitude of force needed to elicit a reflex response, the velocity of contraction, the strength of contraction, and the duration of contraction and relaxation phases. The final section encloses the techniques of eliciting DTR in the upper extremities, trunk, and lower extremities, and the interpretation of these reflexes.

## Introduction

A reflex is an involuntary, unlearned, repeatable response to a specific stimulus that does not require any input from the brain (1). The deep tendon reflex (DTR), also known as a myotatic reflex, is a sequence of lengthening, contraction, and relaxation of a group of muscles. A DTR comprises of a reflex arc, which is a neural pathway that controls a reflex. The reflex arc is made up of five components:

- Receptor: muscle spindle
- Afferent fibre: Ia afferent fibres
- Integration centre: lamina IX of the spinal cord, synapse on the a-motoneurons
- Efferent fibre
- Effector: muscle

The muscle spindle is a receptor within the muscle that detects changes in the length of the muscle. The muscle spindle consists of a noncontractile centre portion and intrafusal muscle fibres which make up the contractile portion. Tapping the tendon will cause stretching of the muscle spindle, activating it, leading to the propagation of an action potential to the spinal cord via afferent Ia fibres through the dorsal horn. In the spinal cord, the afferent nerve fibre synapses with a-motoneurons that supply the agonist muscles and also synapses with an inhibitory neuron that inhibits the antagonistic muscle group. This causes a concomitant relaxation of the antagonistic muscle as the agonist muscle groups contract.

The firing of the afferent fibres (reflecting the sensitivity of the central portion) depends on the length of the intrafusal fibres. The intrafusal fibres are controlled by gamma motoneurons, which are influenced by the cortex, cerebellum and various brainstem nuclei. This forms the suprasegmental control that modulates reflex activity. The higher centres receive information from the muscle spindles. For example, the dorsal spinocerebellar tract conveys the information about the proprioceptive organs from the muscle spindle to the cerebellum. In return, the higher centres modulate the segmental activity through gamma motoneurons. This, in turn, regulates the quality and amount of information received as the ‘sensitivity’ of the central portion depends on the length of the intrafusal fibres.

## A Brief History

Reflexes have been studied for centuries and it begins with Aristotle and Galen. French physiologist, François Magendie in Paris and surgeon-anatomist, Charles Bell in London were among the first to identify the sensory and motor function of spinal nerve roots (2). In the late 18th century, Robert Whytt, Johann Unzer and Prochaska set up the reflex concept (1, 3) while the concept of the ‘reflex arc’ was formulated in the 1830s by Marshall Hall (1790–1857). However, the concept underwent multiple developments and refinements conducted by various physicians in the late 1800s to reach the neurological examination concept that is applied today. Charles Sherrington played a central role in understanding the reflex activity from his vast physiologic investigations in the late 1800s to early 1900s (1, 2).

### Muscle Stretch Reflexes or Deep Tendon Reflexes

In 1859, Silas Weir Mitchell was the first person to appreciate the response elicited by percussed muscles with a hammer. The term ‘reflex’ was not used at that time and the percussing action was performed on muscles instead of the tendons. Jean-Martin Charcot recognised the diagnostic significance of hyperactive and absent muscle stretch reflex; however, this was only studied systematically by Erb and Westphal 16 years later and the findings were published in the *German Archives of Psychiatry and Nervous Diseases*.

### The Tools

#### Percussion Hammers (3, 4)

In 1761, Viennese physician, Leopold Auenbrugger (1722–1809) described the use of percussion on the chest, back and abdomen as an aid to medical diagnosis. This technique is inspired by the thumping of wine casks to determine the level of the fluid.

In 1826, French Pierre Adolphe Piorry (1794–1879) invented a pleximeter, a resonator in the form of small ivory, metal, cedar or river disk, that was placed on the chest and struck with a finger. Two years later, a percussion hammer was devised by a Scottish physician, Sir David Barry (1781–1836) to strike the pleximeter. However, the percussion hammer was not widely adopted.

**Figure 1:**
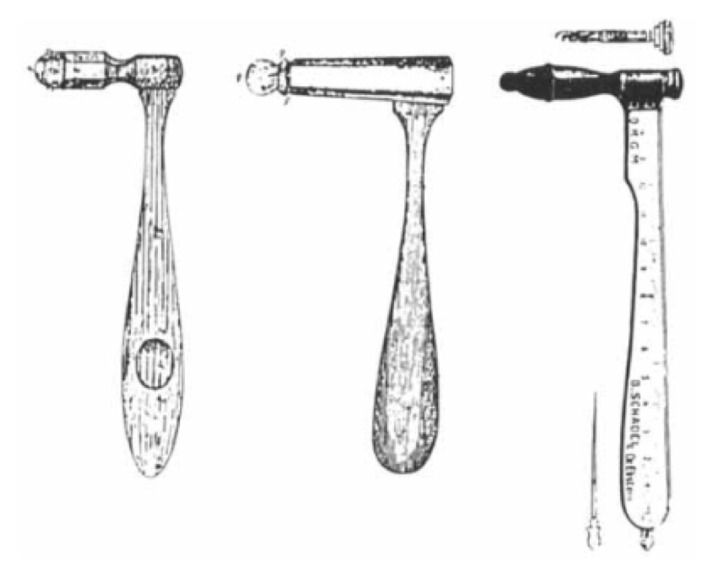
The Wintrich hammer and its modifications

In 1841, a German clinician, Max A Wintrich (1812–1882) introduced a percussion hammer, known as the Wintrich hammer (Figure 1), which was then widely used throughout Europe. The hammer underwent many modifications using different materials, weight and shape. In 1912, the hammer was refurbished by Ebstein into a new model called the ‘reflex and sensibility tester’ for striking the tendons, with a pin on one end.

#### Reflex Hammers (2, 3, 4)

As the practice of testing the DTR grew, so did the development of a variety of percussion hammers. The early percussion hammers were too light and produced inconsistent results. Berliner and Troemner then suggested that an ideal instrument should be heavier to facilitate a brief but forceful percussion without eliciting pain and striking the tendon over a large surface.

The newly refurbished hammers were heavier, and some incorporated a handle and a pointed tip to test cutaneous reflexes, a ruler along the handle, and a pin, brush, or other tools for testing sensation. Some hammers are combined with a long, flexible handle to increase their effectiveness. Tendons were best struck directly, leading to the development of softer rubber striking surfaces and a more refined technique.

#### The Taylor Hammer

Inventor: John Madison Taylor (1855–1931), a neurologist from Philadelphia

Introduced: 1888

Manufacturer: Snowdon (Brothers’ Surgical Instruments Co.) of Philadelphia

Weight: 80 g–140 g

This is the first reflex hammer demonstrated to the Philadelphia Neurological Society on 27 February 1888. The original model was shaped as a flattened cone with a base similar to the striking surface of the ulnar side of the palm, often used to elicit tendon jerk and a rounded apex to elicit biceps jerk (3, 4). It was made of moderately soft rubber and encircled by a metal band, connecting to a rigid straight handle which finished as an open loop. Dr Taylor believed that a rigid handle allowed better control as the elastic handle produced inconsistent force.

**Figure 2:**
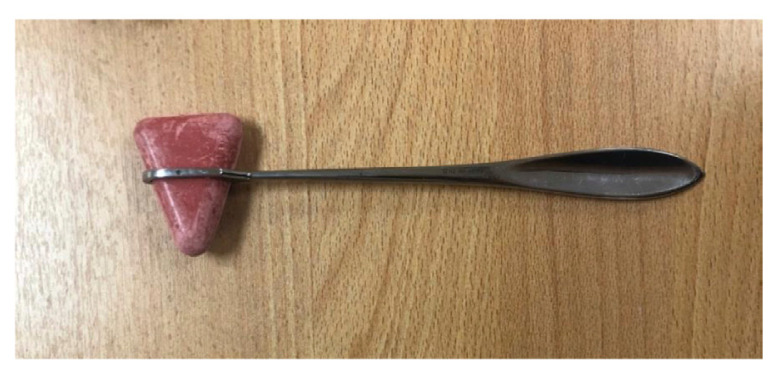
The Taylor hammer

**Figure 3:**
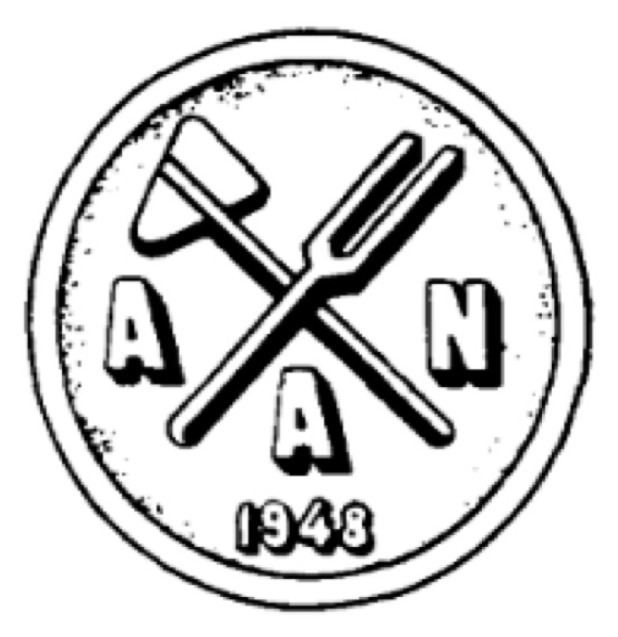
The American Academy of Neurology logo

In 1920, the open-loop handle was replaced with a solid and sharpened tip (Figure 2) to allow for testing of the cutaneous reflexes (4), eliciting chest sounds and percussing the abdomen. *The American Academy of Neurology* incorporated the Taylor hammer into its logo (Figure 3).

#### The Krauss Hammer

Inventor: William Christopher Krauss (1863–1909), a neurologist from Buffalo, New York Introduced: 1894 Manufacturer: G Tiemann Company of New York

**Figure 4:**
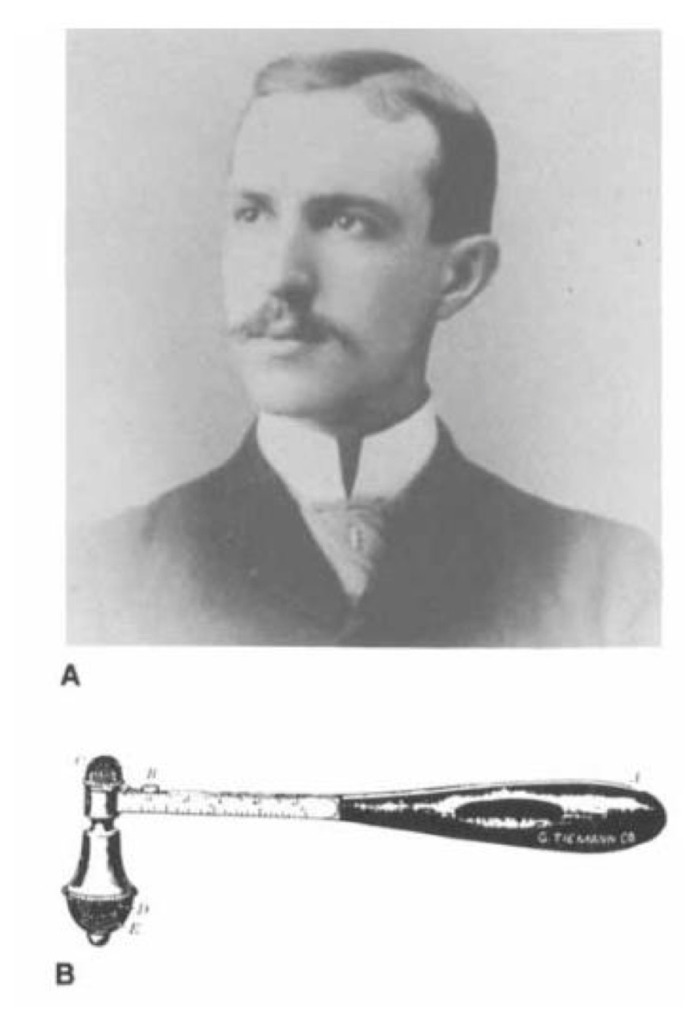
William Christopher Krauss (A). The Krauss reflex hammer (B)

Inspired by the French percussion hammers, the Krauss hammer [Figure 4(b)] has a heavy metallic head fixed to a flattened oval handle with a length of 17 cm (3, 4). This is also one of the earliest hammers to allow for testing sensation and reflexes.

The hammer can be used to test temperature by warming the handle that is made of hard rubber and a metal head that remains cold. There is also a 1.5 cm sharp triangular spearhead under a removable cap and a dull rounded rubber point to examine for anaesthesia or hyperaesthesia. It is also incorporated with a two-point discriminator, a metallic cap to provide a hard surface, and camel’s hair-brush underneath the cap for a soft surface (3, 4).

#### The Troemner Hammer (3, 4)

Inventor: Ernst LO Troemner (1868–1930), German neurologist

Introduced: 1910

Manufacturer: BB Cassel in Frankfurt and Krauth and Company in Hamburg

Weight: > 100 g

**Figure 5:**
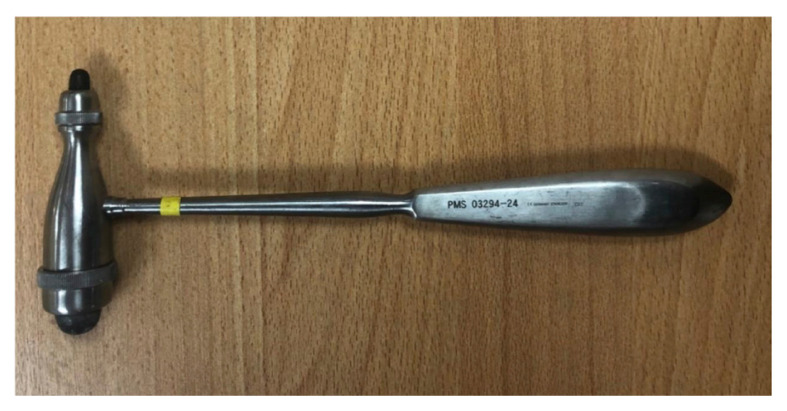
The Troemner hammer

The hammer is all metal with replaceable rubber knobs on both ends of the head (Figure 5) and is based on the older French model. It weighs 100 g and is 22 cm long with a width of 8 cm for the head. The large head is designed for large tendons of extensor surfaces such as patellar, Achilles and triceps reflexes, and also to elicit periosteal and joint reflexes. The smaller head is used for percussion of flexor tendons such as biceps humeri, biceps femoris and semitendinosus. The hammer has a smooth handle that may be used as a tongue blade and a sharpened edge to elicit cutaneous and vascular reflexes. However, Ebstein opposed the usage of the hammer shaft as a tongue blade for hygienic reasons.

#### The Berliner Hammer (3, 4)

Inventor: Bernhard Berliner, German neurologist Introduced: 1910

Manufacturer: Louis and H. Lowenstein, Berlin

**Figure 6:**
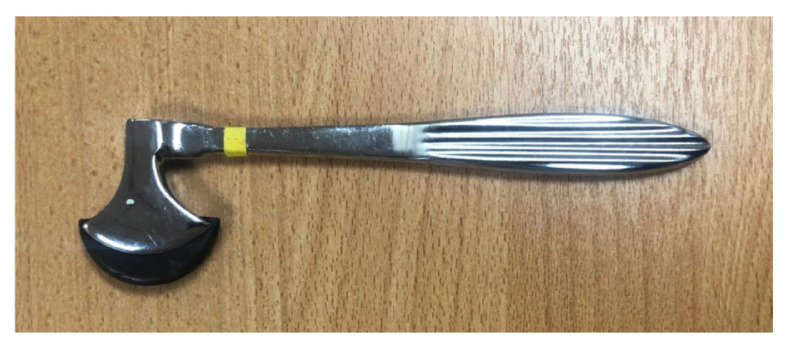
The Berliner hammer

The metal and nickel-plated hammer (Figure 6) has a sufficiently heavy head and a large striking surface covered with rubber. The shape is like a hatchet with a tapered handle to test skin reflexes.

#### The Babinski/Rabiner Hammer (3, 4)

Inventor: Joseph Francois Babinski, French neurologist

Introduced: 1896

**Figure 7:**
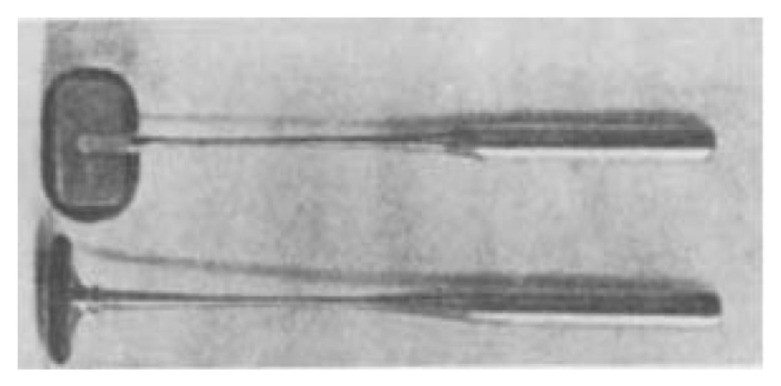
The Babinski reflex hammer

Babinski described two types of hammers (Figure 7) with the same 20 cm–25 cm long nickel-plated steel handle. One of the hammers has a disk encased by a peripheral rubber ring fixed at the end while for the other hammer, the disk is replaced with a rectangular plate in the same plane as the handle for an easier fit into the pocket.

**Figure 8:**
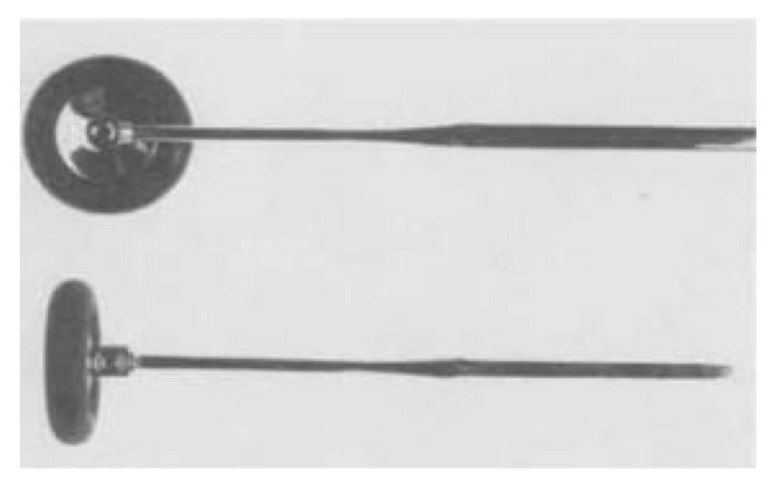
The Rabiner reflex hammer

American neurologist, Abraham Rabiner (1892–1917) modified the Babinski hammer by screwing the head to a shank, allowing the use of the head either parallel or perpendicular to the handle (Figure 8).

#### The Dejerine Hammer (4)

Inventor: Joseph Jules Dejerine (1849–1917)

**Figure 9:**
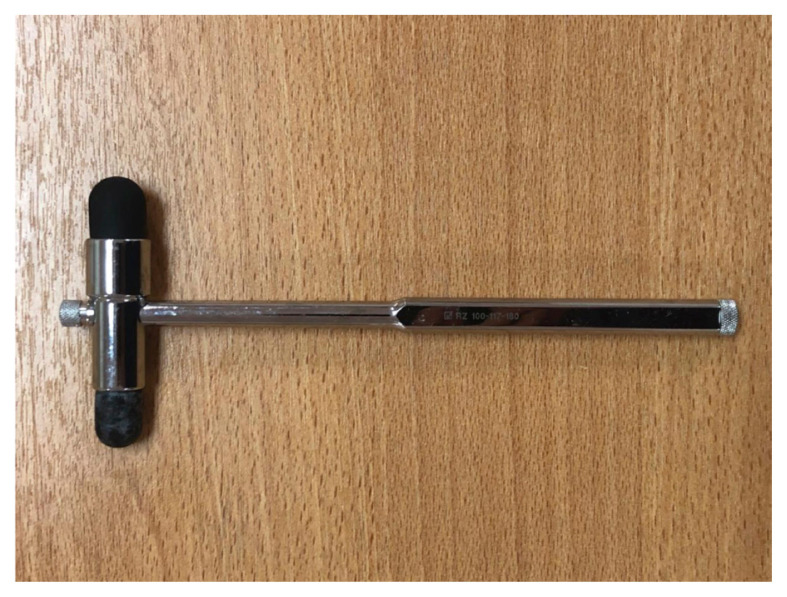
The Dejerine reflex hammer

The hammer (Figure 9) is designed with a blunt handle, and the heads were formed by a rubber cylinder encircled by a metal.

#### The Queen Square Hammer (3, 4)

Inventor: Miss Wintle, head nurse of physiotherapy and radiology at the National Hospital for Nervous Diseases, Queen Square, London

Introduced: 1925

Manufacturer: Messrs Whicker and Blaise of St. James’s Street

**Figure 10:**
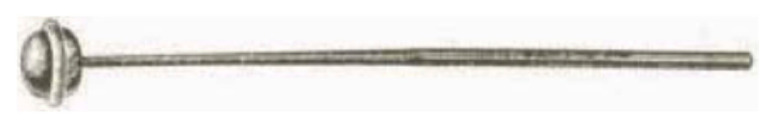
The Vernon percussion hammer (1858) was the precursor of the Queen Square reflex hammer

**Figure 11:**
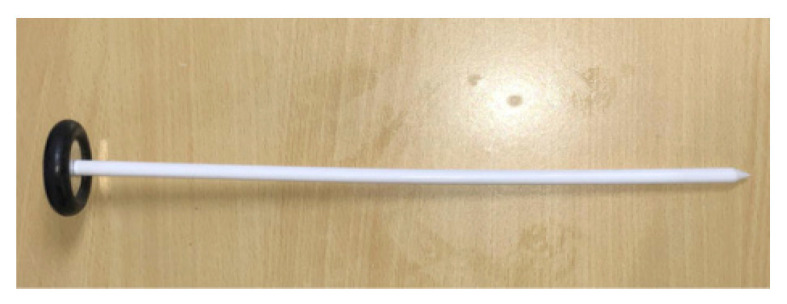
The Queen Square reflex hammer

The hammer was modelled after the Henry Vernon chest percussion hammer (introduced in 1858). The hammer consists of a slender 8-inch-long flexible bamboo cane handle with a 1.5-inch rubber ring fitted brass disk on one end (Figures 10 and 11). The other end is sharpened to elicit cutaneous reflexes. This resulted in a heavy, springy and completely painless hammer.

Marshall and Little (5) conducted a study to examine the peak tap force exerted by the Taylor hammer, the Queen Square hammer and the Babinski hammer. The findings showed that the Taylor hammer exerted the smallest peak tap force due to the lower mass and shorter handle. The peak tap force by the Queen Square hammer elicited a larger reflex response and this could be due to the flexible handle yielding a longer duration tap compared to the rigid handles. Hence, the Queen Square hammer and the Babinski hammer were suggested to be superior for reflex testing.

#### The Techniques

An important feature of a reflex response includes (6):

- Amount of hammer force to obtain the contraction
- Velocity of the contraction
- Strength of the contraction
- Duration of the contraction
- Duration of the relaxation phase
- Response of other muscles that were not tested

Using an instrumented tendon hammer with a force sensor, Zhang et al. (7) found that the reflex torque (output) depends on the tapping force (input). A larger amplitude tapping force (within limits) produces larger amplitude reflex torques. The tapping force threshold is 20 N in normoreflexic patients. This yields a reflex torque of 4 Nm. In hyperreflexic patients, the tapping force threshold is lower, 13 N–15 N resulting in a larger reflex torque of 10 Nm–11 Nm.

These findings were reproduced in a clinical study conducted by Marshall and Little (5) investigating the typical tap forces used by clinicians to assess the patellar reflex. The peak tap forces:

- 0 N–20 N for hyperreflexia
- 21 N–50 N for normoreflexia
- > 50 N for hyporeflexia

A proper technique does not require excessive force (8). Electrophysiological studies of the H-reflex recorded at the soleus muscle showed that an increase in the amplitude of stimulation increases the H-wave. Further increase in the stimulation amplitude resulted in the M-response. When the M-response is maximal, the H-wave vanishes.

This occurs because the large amplitude of electrical current stimulates the afferent Ia fibres and also the thinner α-motor neurons. The action potentials in the α-motor neurons propagate to the spinal cord (antidromic), collide with the action potentials of the evoked reflex response (orthodromic) and cause a partial to complete cancellation effect of the evoked reflex response (9, 10).

On the electromyography studies, the onset latency of DTRs is about 25 msec–45 msec depending on the distance of the stretched muscle from the spinal cord and whether the tendon was tapped, or the muscle was mechanically stretched (10).

In a study of the ankle jerk reflex, the peak torque was 6.3 Nm ± 2.6 Nm for young adults whereas elderly subjects showed a peak torque of 3.6 Nm–4.3 Nm. The half contraction time in the young adults and the elderly was 27.9 msec–28.9 msec. The half relaxation time for the young adults was 30.0 msec–43.8 msec and 36.4 msec–44.3 msec in the elderly (11).

### Reflexes of the Upper Extremity (12, 13, 14)

#### Biceps Reflex (Figure 12)

Striking point: Examiner’s finger over the biceps tendon

Myotome: C5/C6

Peripheral nerve: Musculocutaneous nerve

Effector: Biceps

Technique:

- Place the patient’s forearm in slight pronation, midway between flexion and extension
- Palpate the biceps tendon, stretching it by pushing it slightly towards the patient’s wrist
- Strike the examiner’s finger with the pointed end of the Taylor hammer
- Observe the biceps muscle for contraction with slight flexion of the elbow

#### Brachioradialis Reflex (Radial Periosteal Reflex) (Figure 13)

Striking point: Examiner’s finger at 10 cm above the wrist, proximal to the styloid process of the radius

Myotome: C6

Peripheral nerve: Radial nerve

Effector: Brachioradialis muscle

Technique:

- Place the patient’s forearm in a semi-flexed and semi-prone position
- Palpate the brachioradialis tendon at the junction between the middle and distal thirds of the forearm (the muscle becomes tendinous around the mid-forearm)
- Strike the hammer on the examiner’s finger
- Observe for flexion of the elbow with variable supination

If there is associated flexion of the fingers and the wrist, the reflex is exaggerated. An inverted brachioradialis reflex is demonstrated when there is an absence of flexion and supination of the elbow with the presence of flexion of the finger and the wrist flexors.

#### Triceps Reflex

Striking point: Triceps tendon above its insertion on the olecranon process of the ulna

Myotome: C7/C8

Peripheral nerve: Radial nerve

Effector: Triceps muscle

Technique:

- Support the patient’s arm midway between flexion and extension. Allow the elbow to flex about 40°
- Strike the tendon with the broad end of the Taylor hammer
- Observe the triceps muscle for contraction with an extension of the elbow

An inverted triceps jerk is demonstrated when there is flexion of the elbow joint on striking the triceps tendon. It indicates an injury to the afferent part of the reflex arc.

#### Finger Flexor Reflex

Striking point: Examiner’s fingers on the palmar surface of the patient’s fingers

Myotome: C8/T1

Peripheral nerve: Median and ulnar nerves

Technique:

- Place the patient’s hand on a stable surface in a supine position
- Place the examiner’s fingers against the palmar surface of the patient’s fingers
- Strike the dorsum of the examiner’s fingers lightly with the reflex hammer
- Observe for flexion of the patient’s fingers and distal phalanx of the thumb

#### Scapulohumeral Reflex (15)

Striking point: Tip of the scapula at the vertebral body

Peripheral nerve: Dorsal scapular nerve

Myotome: C4/C5

Effector: Rhomboid muscle

Technique:

- Tap the tendon hammer over the spine of the scapula at the tip in a caudal direction
- Observe for retraction of the scapula with associated adduction of the humerus

A hyperactive scapulohumeral reflex is demonstrated only when there is an elevation of the scapula or abduction of the humerus.

#### Deltoid Reflex (Figure 14)

Striking point: Examiner’s finger over the insertion point of the deltoid muscle on the lateral surface of the humerus

Peripheral nerve: Axillary nerve

Myotome: C5/C6

Effector: Deltoid muscle

Technique:

- Palpate the deltoid tendon at the junction of the upper and middle third on the lateral aspect of the humerus
- Strike the finger with a reflex hammer
- Observe for slight abduction of the upper arm.

#### Pectoralis Reflex (Figure 15)

Striking point: Examiner’s finger over the deltopectoral groove

Peripheral nerve: Medial and lateral pectoral nerves

Myotome: C5–T1

Effector: Pectoralis muscle

Technique:

- Place the patient’s arm in mid-position between abduction and adduction
- Palpate the tendon of the pectoralis major muscle at the deltopectoral groove
- Strike the finger with a reflex hammer
- Observe for adduction and slight internal rotation of the arm at the shoulder

This reflex is absent in normal individuals. A hyperactive pectoralis reflex is an indication of an upper cervical cord injury.

#### Latissimus Dorsi Reflex

Striking point: Examiner’s finger over latissimus dorsi tendon

Peripheral nerve: Thoracodorsal nerve

Myotome: C6–C8

Effector: Latissimus dorsi muscle

Technique:

- Place the patient in a prone position with arms abducted and slightly externally rotated
- Palpate the latissimus dorsi tendon at the intertubercular groove of the humerus
- Strike the finger with a reflex hammer
- Observe for the abduction and slight shoulder internal rotation

#### Clavicle Reflex (Figure 16)

Striking point: Lateral aspect of the clavicle

Technique:

- Tap the lateral aspect of the clavicle
- Observe for a contraction of various muscle groups of the upper limbs or any asymmetry of the reflex on either side of the upper limb

This is used to compare the reflex activity of both upper limbs. The presence of a hyperactive reflex indicates a reflex spread.

#### Truncal Reflexes (12, 13, 14)

Reflexes from the trunk are minimally obtained or absent in normal individuals.

#### Costal Periosteal Reflex

Striking point: Xiphoid process, lower rib margins

Peripheral nerve: Upper intercostal nerves Myotome: T5–T9

Technique:

- Tap the lower rib margins or the xiphoid process
- Observe for a contraction of the upper abdominal muscles and slight movement of the umbilicus towards the side of the stimulation

#### Abdominal Muscle Reflex (Deep Abdominal)

Striking point: Muscles overlying abdominal wall Peripheral nerve: Intercostal nerves (anterior divisions of T5–T12), ilioinguinal and iliohypogastric nerves

Technique:

- Stretch the abdominal wall muscles by pressing down with a tongue blade or index finger
- Strike the tongue blade or finger with a reflex hammer
- Observe for a contraction of the abdominal muscle and deviation of the umbilicus towards the stimulus

The deep abdominal reflexes were described by Gerhardt in 1895. In normal individuals, this reflex is minimally present. If the deep abdominal reflexes are exaggerated but the superficial abdominal reflexes are absent, this suggests a corticospinal tract lesion above T6.

#### Iliac Reflex

Striking point: Iliac crest

Peripheral nerve: Lower intercostal nerves (T10–T12)

Technique:

- Tap the iliac crest with a reflex hammer
- Observe for contraction of the lower abdominal muscles

#### Symphysis Pubis Reflex

Striking point: Symphysis pubis

Peripheral nerve: Lower intercostal, ilioinguinal, and iliohypogastric nerves

Myotome: T11–T12 and upper lumbar segments

Technique:

- Place the patient in a recumbent position
- Tap the symphysis pubis, 1.5 cm–2 cm from the midline for a unilateral stimulus
- Observe the lower abdominal muscles for contraction and downward movement of the umbilicus. This is the ‘upper response’

If the reflex is exaggerated, a ‘lower response’ or puboadductor reflex occurs. The ipsilateral adductor muscles contract with some hip flexion.

### Reflexes of the Lower Extremity (12, 13, 14)

#### Patellar Reflex aka Knee Jerk (Erb and Westphal, 1870–1871) (Figure 17)

Striking point: Patellar tendon

Myotome: L3/L4

Peripheral nerve: Femoral nerve

Effector: Quadriceps femoris muscle

Technique:

- In a supine patient, place the knee and hip in a partially flexed position by placing one hand beneath the knee to stretch the tendon while ensuring the limb is relaxed
- In a sitting patient, have the legs dangling with the heels resting on the floor. Another technique is to have the patient sit with one leg crossed over the other and tapping the uppermost leg
- Palpate the patellar ligament
- Tap the tendon with the broad end of the Taylor hammer

If the reflex is exaggerated, the response may be obtained by tapping the tendon just above the patella (suprapatellar reflex). This is done by placing the examiner’s index finger on the upper border of the patella and tapping the finger to push down the patella. In a reflex spread, the knee extension is accompanied by adduction of the hip. An inverted patellar reflex occurs with contraction of the hamstrings and knee flexion (femoral nerve lesion). The Westphal’s sign is the absence of patellar reflex.

This reflex was first described between 1870 and 1871 by Wilhelm Erb, a German neurologist. The findings were published in the *German Archives for Psychiatry and Nervous Disease* in 1875 in collaboration with Carl Westphal. Erb correctly explained the reflex to be mediated by a reflex arc, using the term ‘Patellarsehnenreflex’, which means patellar tendon reflex. Later on, William Gowers from London coined the term ‘knee-jerk’ in 1881. Sherrington (1891–1892) and Dutch neurologist, Jan van Gijn confirmed that the knee-jerk is a true spinal reflex (1, 2, 3).

#### Achilles Reflex or Ankle Jerk

Striking point: Achilles tendon

Myotome: S1/S2

Peripheral nerve: Tibial nerve

Effector: Gastrocnemius muscle, soleus muscles

**Figure 18:**
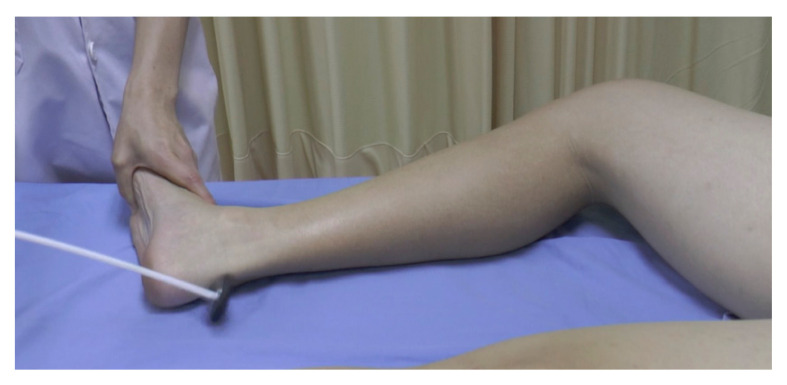
Frog-leg position

**Figure 19:**
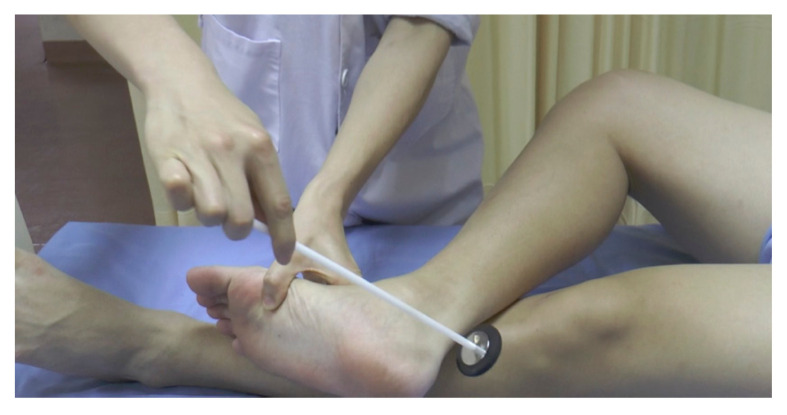
Figure four position

Technique:

- In a patient who is seated or lying on a bed, place the thigh in a moderately abducted and externally rotated position with the knee flexed. If the patient is supine, place the patient’s legs into a frog-leg position (Figure 18) with knees apart and ankles close together. Another position is to cross the examined leg over the shin or ankle (figure four position, Figure 19)
- Passively dorsiflex the ankle to about 90º to stretch the tendon
- Palpate the Achilles tendon
- Tap the tendon at the level of the ankle bone with the broad end of the Taylor hammer

Babinski introduced the methods where the patient kneels on a chair with the feet projecting at right angles. It is useful to compare reflex activity on both sides.

In a hyperactive reflex, tapping other areas of the sole (medioplantar reflex) will elicit the reflex. With reflex spread, striking the Achilles tendon may cause knee flexion. Paradoxical ankle reflex occurs when tapping the anterior aspect of the ankle produces reflex. It is important to note that studies by O’Keefe et al. (16) and Schwartz et al. (17) showed that the plantar strike technique is a more reliable method in elderly patients.

Technique*:*

- Place the examiner’s fingers on the patient’s forefoot with slight tension to the dorsiflex of the foot
- Tap the examiner’s fingers
- Observe for plantarflexion

The Achilles reflex was also described by Erb and Westphal in an article published in the *German Archives for Psychiatry and Nervous Disease* in 1875 (1, 2, 3).

The following reflexes are less significant as they may be difficult to be elicited in normal patients. These reflexes are significant with unilateral absence or the presence of exaggeration which suggest corticospinal tract disease.

#### Adductor Reflex (Figure 20)

Striking point: Medial epicondyle of the femur

Peripheral nerve: Obturator nerve

Myotome: L2–L4

Effector: Adductor muscle

Technique:

- Place the thigh in slight abduction
- Tap over the medial epicondyle of the femur or medial condyle of the tibia
- Observe for contraction of the adductor muscles with inward movement of the limb

An exaggerated response is present with crossed or bilateral adduction suggesting corticospinal tract disease.

#### Internal Hamstring (Semimembranosus and Semitendinosus or Posterior Tibiofemoral) Reflex (18) (Figure 21)

Striking point: Semimembranous or semitendinous tendons at the insertion on the tibia

Peripheral nerve: Tibial portion of the sciatic nerve

Myotome: L5

Effector: Hamstring muscles

Technique:

- In a supine patient, slightly flex the hip and externally rotate and flex the knee, with the leg supported by one of the examiner’s hands
- Palpate the tendons on the medial posterior aspect of the knees
- Strike the fingers with a reflex hammer
- Observe for hip extension and knee flexion with internal rotation

It is mediated primarily by the tibial portion of the sciatic nerve (L5) and is the only DTR useful in evaluating suspected L5 radiculopathy.

#### External Hamstring Reflex (Biceps Femoris or Posterior Peroneofemoral) (Figure 22)

Striking point: Biceps femoris tendon or head of the fibula

Peripheral nerve: Tibial portion of the sciatic nerve

Myotome: S1

Effector: Hamstring muscles

Technique:

- In a patient who is sitting, recumbent, or lying on the opposite side, moderately flex the knee
- Palpate the tendon at the posterolateral aspect of the knee and tap on the fingers
- Observe for knee flexion

This reflex helps to determine if an absence of ankle jerk is due to peripheral neuropathy (preserved external hamstring reflex) or due to radiculopathy (absent lateral hamstring reflex).

#### Tensor Lata Reflex

Striking point: Near the anterior superior iliac spine

Peripheral nerve: Superior gluteal nerve

Myotome: L4–S1

Effector: Tensor fascia lata (TFL)

Technique:

- In a recumbent patient, tap the origin on the TFL near the anterior superior iliac spine
- Observe for slight abduction of the thigh

#### Gluteal Reflex

Striking point: Lower portion of the sacrum

Peripheral nerve: Inferior gluteal nerve

Myotome: L5–S2

Effector: Gluteus maximus muscle

Technique:

- In a recumbent patient, position the patient by shifting the weight to the opposite side with moderate flexion of the ipsilateral thigh. Alternatively, the patient can be in a prone position
- Tap the lower portion of the sacrum or the posterior aspect of the ilium near the insertion of the gluteus maximus
- Observe for a contraction of the gluteus maximus and extension of the thigh

#### Tibialis Posterior Reflex

Striking point: Tibialis posterior tendon behind the medial malleolus

Peripheral nerve: Tibial nerve

Myotome: L5–S1

Effector: Tibialis posterior muscle

Technique:

- In a prone patient, slightly flex the patient’s knee and support the leg
- Place the foot beyond the edge of the bed with slight eversion
- Tap the tibialis posterior just above and behind the medial malleolus
- Observe for foot inversion

#### Peroneal (Tibialis Anterior) Reflex

Striking point: Fingers over the distal first and second metatarsal bones

Peripheral nerve: Deep and superficial peroneal nerve

Myotome: L4–S1

Effector: Tibialis anterior, peronei brevis, and peronei longus muscles

Technique:

- Place the patient’s foot in a plantarflexed and inverted position
- With the examiner’s fingers placed firmly on the distal first and second metatarsal bones, tap the fingers
- Observe for eversion and dorsiflexion of the foot

#### Jaw Jerk (Morris Lewis, 1885)

Striking point: Examiner’s finger on the tip of the jaw

Afferent: Motor root of V3 → Mesencephalic nucleus

Peripheral nerve: Motor nucleus → V3 division

Effector: Masseter muscle

Technique:

- Have the patient relax and hang the jaw loosely with the mouth slightly open
- The examiner rests a finger across the tip of the jaw and strikes downward with a tendon hammer
- Observe for upward movement of the jaw

This reflex was described as ‘chin reflex’ by Philadelphia neurologist, Morris Lewis in 1885. An exaggerated response implies bilateral disease above the pons (pseudobulbar palsy).

## NINDS Myotatic Reflex Scale Versus the British Scale System (19, 20, 21)

The Clinical Neuroscience Programme, Division of Intramural Research, National Institute of Neurological Disorders and Stroke (NINDS) has adopted the use of NINDS myotatic reflex scale as the standard scale to assess the DTR. The reliability of the NINDS myotatic reflex scale to be adopted as the universal scale has been confirmed by Litvan et al. (20). Another scale that can be used is the British scale. The British scale is a 5-point scale that distinguishes sustained clonus (5+) from intermittent clonus (4+). The scales are shown in Table 1.

## Interpretation

The DTR depends on the integrity of the upper motor neuron and lower motor neuron. An exaggerated DTR suggests an upper motor neuron disease and a reduction or loss of DTR suggests a lower motor neuron disease. Certain patients may have absent or exaggerated reflexes. However, on their own, these findings do not confirm a neurological disease. They are only significant if the reflex amplitude is asymmetrical, unusually brisk, or absent compared to other spinal levels and if they are associated with other neurological signs to indicate either an upper motor neuron or lower motor neuron disease.

Other findings such as reflex spread is defined as the increase in the reflexogenic zone. The reflex tested is present but is accompanied by a response in other muscles. For example, the biceps reflex is obtained by tapping the clavicle and is accompanied by flexion of the fingers. An inverted reflex is seen when the reflex tested is absent but creates a contraction in other muscles. An inverted brachioradialis reflex is seen when the afferent limb of the reflex arc is impaired. There is an absence of supination and flexion of the elbow with twitching or flexion of the hand and fingers.

A video (https://youtu.be/Fz4kAa4Wj_g) has been produced to demonstrate the examination techniques described in this article.

## Conclusion

The DTR remains an important aspect of the neurological exam despite the use of modern imaging. The reflex hammers have undergone multiple modifications throughout the years to produce a heavy, high-quality tool with a flexible handle to give a crisp blow to effectively stretch the tendon. More importantly, clinicians should master the correct technique to perform the art of eliciting DTR as this provides valuable information on the condition of the nervous system. The use of correct tools and techniques increases the validity of the findings. A good understanding of the physiology of the DTR allows precise interpretation of the clinical findings.

## 

## Figures and Tables

**Figure 12:**
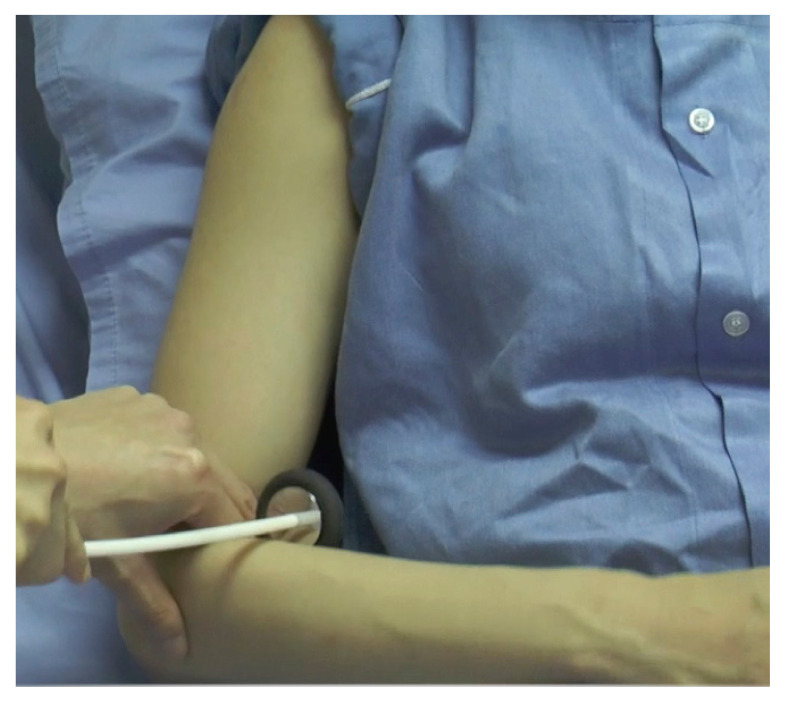
Striking the biceps tendon

**Figure 13:**
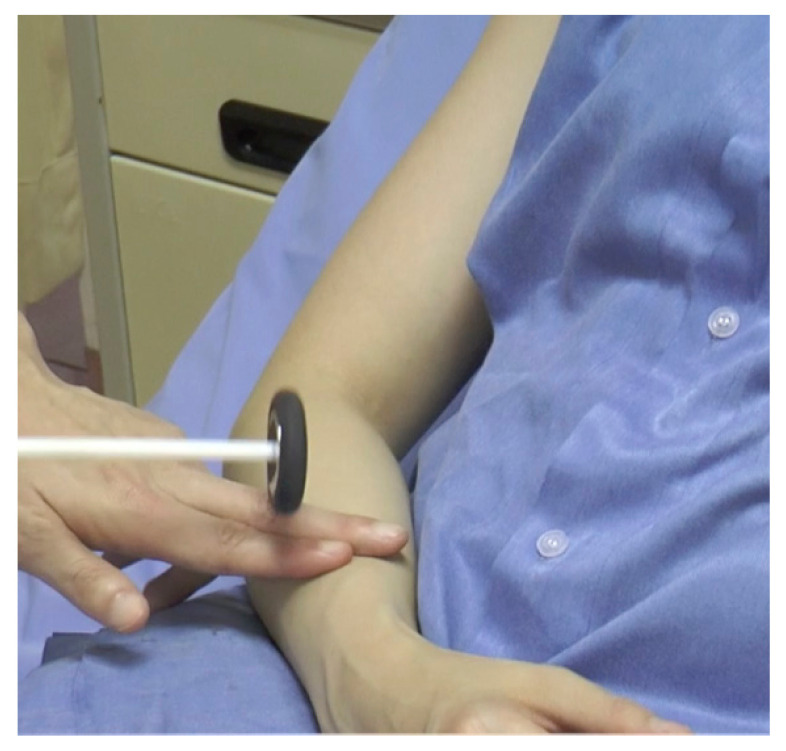
Striking the brachioradialis tendon

**Figure 14:**
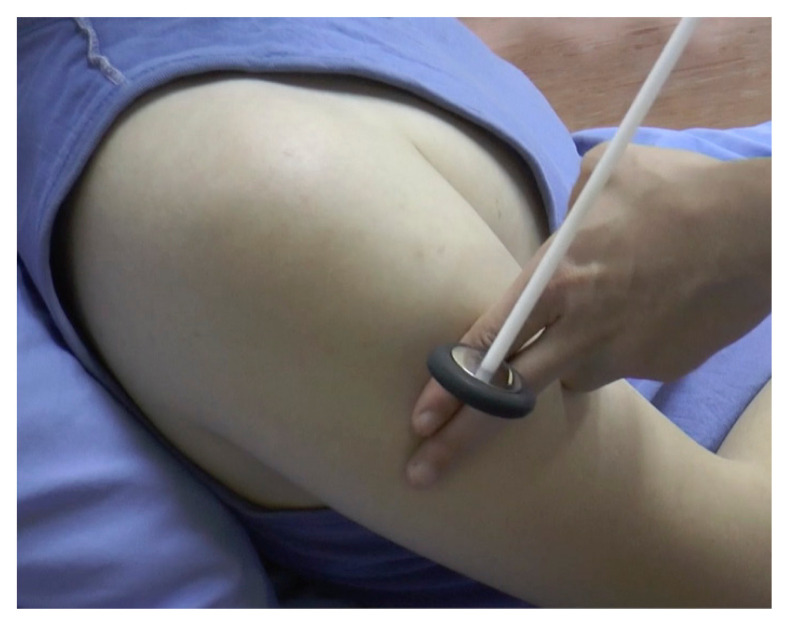
Striking the deltoid tendon

**Figure 15:**
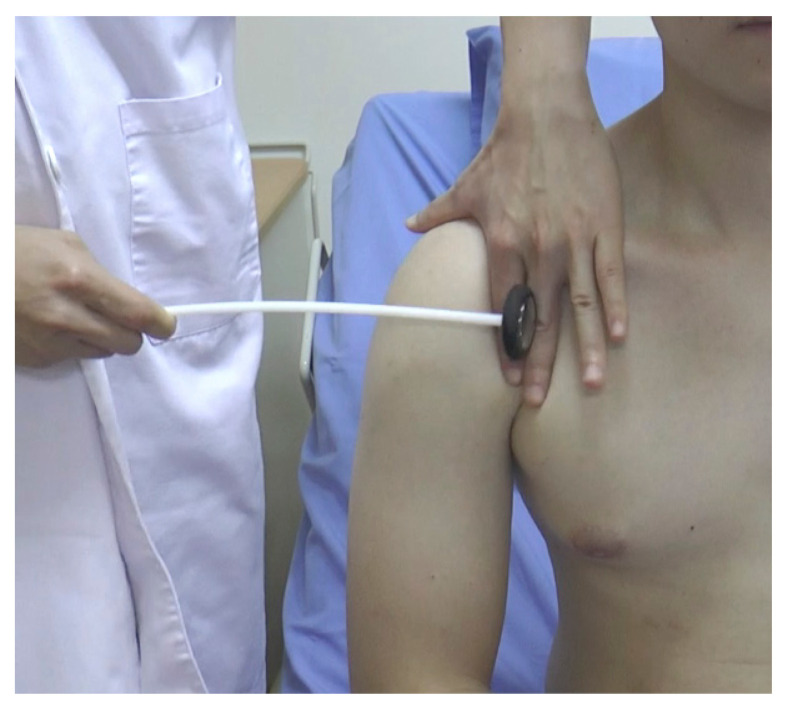
Striking the tendon of the pectoralis major muscle at the deltopectoral groove

**Figure 16:**
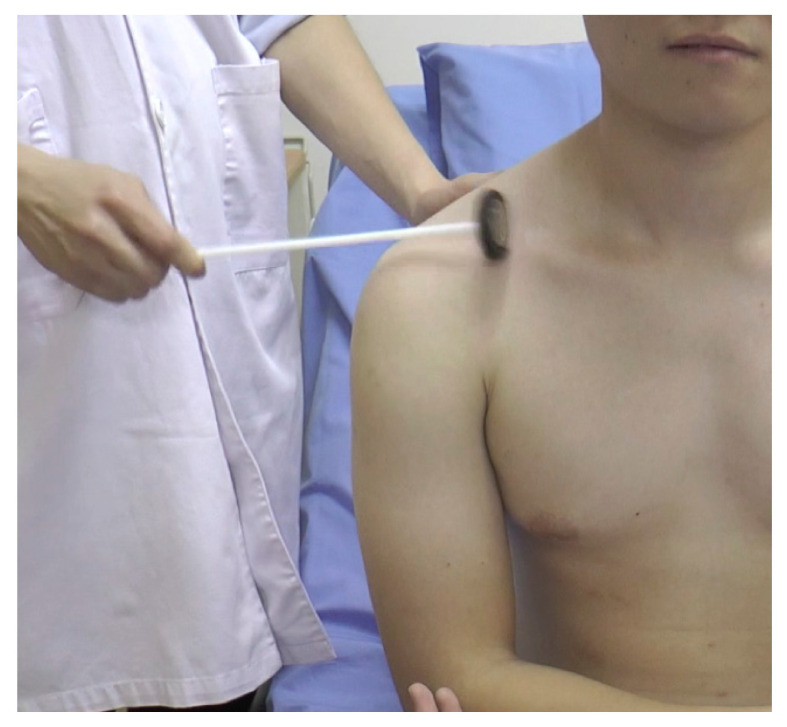
Striking the lateral aspect of the clavicle

**Figure 17:**
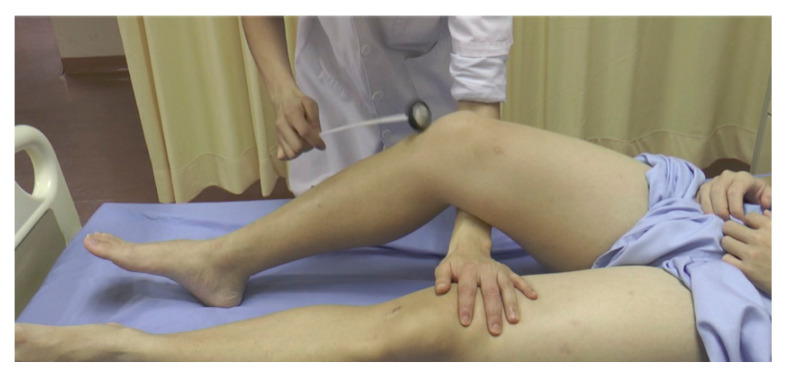
Supporting the tested limb and striking the patellar tendon

**Figure 20:**
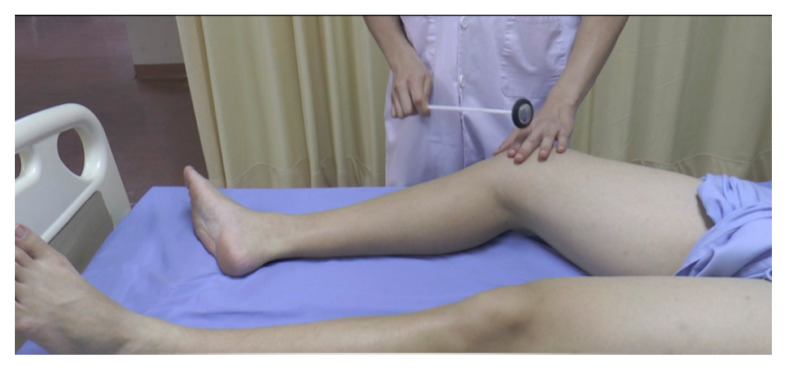
Striking the medial epicondyle of the femur

**Figure 21:**
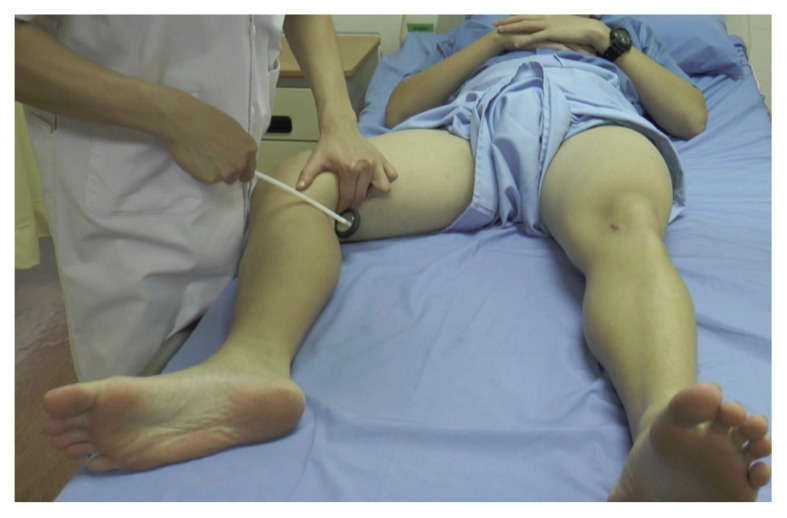
Striking the semimembranous or semitendinous tendons

**Figure 22:**
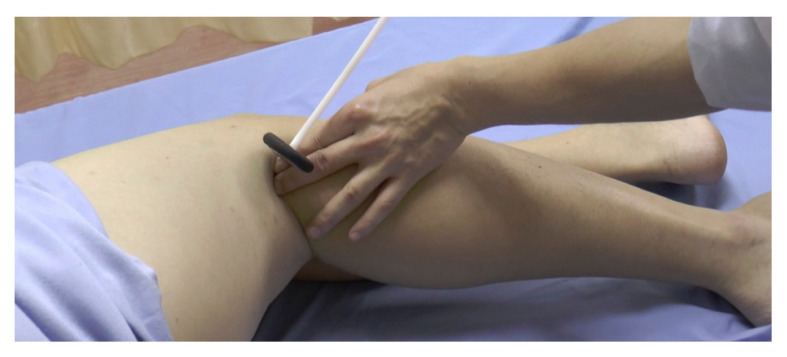
Striking the biceps femoris tendon

**Table 1:** Comparison between the NINDS myotatic reflex scale and British scale

Scale	NINDS scale (0–4)	Scale	British scale (0–5+)	Description
0	Reflex absent	0	Reflex absent	No perceptible response
1	Reflex small, less than normal, includes a trace response or a response brought out only with reinforcement	1+	Reflex small, less than normal, includes a trace response or a response brought out only with reinforcement	Contraction barely perceptible but can be enhanced with Jendrassik manoeuvre
2	Reflex in the lower half of a normal range	2+	Brisk, within the median normal range	Contraction perceptible
3	Reflex in the upper half of a normal range	3+	Reflex enhanced, high normal or hyperreflexia	Vigorous contraction is apparent from across the room
4	Reflex enhanced, more than normal, includes clonus if present, which optionally can be noted in an added verbal description of the reflex	4+	Reflex enhanced, more than normal, includes intermittent clonus	Reflex ‘beats’ multiple times at the end of the arc. The only reflex that is always abnormal is clonus
		5+	Sustained clonus	

Notes: The ‘+’ after the number is to distinguish from muscle testing. The Jendrassik manoeuvre is a reinforcement technique, whereby the patient attempts to pull the hands apart with fingers hooked together. The effects last 1 sec–6 sec and is maximal for only 300 msec
